# Dynamic regulation of the transcriptome and proteome of the equine embryo during maternal recognition of pregnancy

**DOI:** 10.1096/fba.2022-00063

**Published:** 2022-10-18

**Authors:** Alba Rudolf Vegas, Giorgia Podico, Igor F. Canisso, Heinrich Bollwein, Thomas Fröhlich, Stefan Bauersachs, Carmen Almiñana

**Affiliations:** ^1^ Functional Genomics Group Institute of Veterinary Anatomy, Vetsuisse‐Faculty, University of Zurich Lindau (ZH) Switzerland; ^2^ Department of Veterinary Clinical Medicine, College of Veterinary Medicine University of Illinois Urbana Champaign Urbana Illinois USA; ^3^ Clinic of Reproductive Medicine, Department for Farm Animals, Vetsuisse‐Faculty University of Zurich Zurich Switzerland; ^4^ Gene Center, Laboratory for Functional Genome Analysis Munich Germany

**Keywords:** conceptus, embryo, Equus caballus, mare, maternal recognition of pregnancy, miRNA, proteomics, transcriptomics

## Abstract

During initial maternal recognition of pregnancy (MRP), the equine embryo displays a series of unique events characterized by rapid blastocyst expansion, secretion of a diverse array of molecules, and transuterine migration to interact with the uterine surface. Up to date, the intricate transcriptome and proteome changes of the embryo underlying these events have not been critically studied in horses. Thus, the objective of this study was to perform an integrative transcriptomic (including mRNA, miRNAs, and other small non‐coding RNAs) and proteomic analysis of embryos collected from days 10 to 13 of gestation. The results revealed dynamic transcriptome profiles with a total of 1311 differentially expressed genes, including 18 microRNAs (miRNAs). Two main profiles for mRNAs and miRNAs were identified, one with higher expression in embryos ≤5 mm and the second with higher expression in embryos ≥7 mm. At the protein level, similar results were obtained, with 259 differentially abundant proteins between small and large embryos. Overall, the findings demonstrated fine‐tuned transcriptomic and proteomic regulations in the developing embryo associated with embryo growth. The identification of specific regulation of mRNAs, proteins, and miRNAs on days 12 and 13 of gestation suggested these molecules as pivotal for embryo development and as involved in MRP, and in establishment of pregnancy in general. In addition, the results revealed new insights into prostaglandin synthesis by the equine embryo, miRNAs and genes potentially involved in modulation of the maternal immune response, regulation of endometrial receptivity and of late implantation in the mare.

## 1 INTRODUCTION

Early pregnancy in equine differs from other mammalian species in several unique features, such as an extended period in the oviduct,^1^ selective embryonic passage from oviduct to the uterus,^2^, ^3^ a spherical migrating embryo (embryo and its associated membranes)^4^, ^5^ enveloped by a glycoprotein embryonic capsule,^6^ the formation of the endometrial cups producing equine chorionic gonadotropin (eCG),^7^ and very late implantation.^8^

The early embryonic development in the horse takes place in the oviduct until entering the uterus 144 h (day 6–6.5) after ovulation in the late morula or early blastocyst stage.^1^, ^5^ Around day 8 post‐ovulation, the zona pellucida disintegrates completely^5^ and the blastocyst is surrounded by an acellular glycoprotein capsule starting 7 days post‐ovulation^6^ until day 23 post‐ovulation.^5^ This embryonic capsule is of a vital importance for embryo survival during this time^9^ and maintains the equine embryo in a spherical shape. Approximately at day 9 post‐ovulation, the equine embryo starts to migrate through the uterus with maximum mobility between days 10 and 14 until it gets fixed at the base of one of the uterine horns around day 16.^4^ During this period, the embryo signals its presence to the endometrium to trigger endometrial and ovarian signaling cascades leading to sustained progesterone production by the corpus luteum (CL), which is essential for maintaining pregnancy and further embryonic development during the first trimester.^10^ However, the exact embryo‐derived signal(s) for MRP remain(s) to be elucidated in the horse.^11^ It has been hypothesized that a combination of mechanical stimuli coupled with the secretion of a variety of molecules (e.g., 17‐α‐hydroxyprogesterone,^12^ estrogens,^13^ prostaglandins (PGs),^14^ interferons^15^) during the migration phase is responsible for MRP in the mare.^16^ This hypothesis is partially supported because none of the known molecules secreted by the equine embryo has been confirmed to be the sole signal for MRP.

Therefore, to identify signals of MRP in the mare, different transcriptomic and proteomic approaches have been carried out. On the endometrium side, transcriptomic gene expression studies on whole endometrial biopsies using microarrays or RNA‐sequencing (RNA‐seq) have been performed.^16^, ^17^, ^18^, ^19^, ^20^, ^21^, ^22^ Moreover, two spatial transcriptomics studies have focused on specific alterations of the different endometrial compartments (luminal epithelium [LE], glandular epithelium [GE], and stroma [ST]) by combining laser capture microdissection (LCM) and low input RNA‐seq.^23^, ^24^ On the embryo side, only a few studies at the mRNA level have been carried out.^19^, ^25^, ^26^ Recently, one study also analyzed miRNAs in the uterine fluid as potentially key players in MRP in horses.^19^

Additionally, proteomic‐based studies of the embryo and/or the surrounding uterine fluid have also been performed.^27^, ^28^, ^29^ Furthermore, the equine embryonic secretome in the blastocyst fluid and during in vitro culture of the embryo for a short period has also been analyzed.^30^ All these studies have provided valuable insights into specific signaling pathways that appear to be involved in the regulation of embryo development and establishment of pregnancy but could not clarify the identity of an embryo‐derived signal leading to MRP in the mare.

Considering the unique features of the equine embryo and current knowledge about MRP in the species, we hypothesized that rather a complex set of molecules than a single signal is mediating MRP in the horse. Therefore, we performed an integrative approach by combining transcriptomic (including protein‐coding RNAs [mRNAs], microRNAs [miRNAs], and other small non‐coding RNAs) and proteomic analysis of embryos collected on days 10–13 after ovulation to gain new insights into the potential embryo‐derived signal(s) contributing to MRP in the horse. Moreover, the resulting data was integrated with results from a concurrent study analyzing the endometrial changes in pregnant and non‐bred cyclic mares.^23^ Piecing together the maternal and embryo signals will help integrate the precise signaling that contributes to establishing the pregnancy state and leads to a successful pregnancy in the mare.

## 2 MATERIALS AND METHODS

### 2.1 Animal trial and embryo collection

All the animal experiments were performed with the revision and permission of the Institutional Animal Care and Use Committee of the University of Illinois Urbana‐Champaign (protocol #16129). Embryo were collected from 10 light breed mares during the physiologic breeding season of the northern hemisphere in 2018. The mares were housed on pasture at the Veterinary Medical Research Farm of the University of Illinois Urbana‐Champaign (Illinois, USA). The cycles of each mare were randomly assigned to one of the four experimental pregnancy days,^10^, ^11^, ^12^, ^13^ and post‐ovulation. Follicular and CL development and ovulation (day 0) were monitored with a portable ultrasound machine (Ibex® EVO® E.I. Medical Imaging) coupled with a 7.5 MHz linear transducer.

Mares were assessed by ultrasonography every other day. If a CL was detected, mares received prostaglandin F2 alpha to return to estrus. When a follicle of at least 30 mm in diameter was detected, mares were examined daily until the detection of a pre‐ovulatory follicle (≥35 mm, in the presence of endometrial edema). Ovulation was hastened with an intramuscular (i.m.) injection of 1.8 mg of deslorelin acetate (SucroMateTM Equine; Thorn BioScience L.L.C.), a GnRH analog (day −2). Mares were inseminated 24 h later (day −1) with 2 billion progressively motile sperm from the same fertile stallion. Uterine lavage was performed 6 h after breeding with Ringer's Lactate Solution (RLS; Lactated Ringer's Injection, USP; Hospira). Twenty International Units (IU) of Oxytocin (VET ONE; Bimeda‐MTC Animal Health Inc.) were administered i.m. immediately after uterine lavage as well as during the next day (2–3 times per day) to avoid intrauterine fluid accumulation. The day when ovulation was detected by transrectal ultrasonography was designated as day 0. Transrectal ultrasonography was performed to detect the presence and to measure the size of the embryonic vesicle on days 10–13 or until sampling. Sampling was carried out on days 10, 11, 12, or 13 post‐ovulation. One hundred milliliters of phosphate‐buffered saline (Corning cellgro) were inserted in the uterus of each mare using an endotracheal tube for small ruminants (DEE Veterinary Products). The uterus was transrectally massaged, and the fluid was recovered into 50 ml conical tubes. After checking the presence of an embryo, the small volume uterine flush was then processed and stored accordingly for a concurrent study. If no conceptus was recovered in the small‐volume uterine flush, transcervical uterine flushing was performed with 1.5–2 L of RLS, repeating this procedure 1–3 times to recover an embryo. The fluid was recovered directly in sterile glass bottles and filtered through an embryo filter (Minitube). The recovered embryos were measured, photographed, immediately snap‐frozen in liquid nitrogen, and stored at −80°C until further analysis. Immediately after sampling, mares received a dose of a synthetic prostaglandin F2α (sodium cloprostenol, 250 μg, i.m., Estrumate®; Merck Animal Health) to induce luteolysis and return to estrus.

### 2.2 Transcriptomic analysis

#### 2.2.1 Isolation of total RNA

Following the manufacturers' recommendations for the mirVana™ PARIS™ kit (Ambion, Life Technologies Corporation), total RNA (including small RNAs) and proteins were isolated from each embryo sample. Only embryos ≤11 mm were used, and two embryos from day 13, broken during the uterine flush (no measurement of size available, probably larger than 15 mm in diameter) due to limitations for the sample size in the lysis protocol used for isolation of RNA and protein. In total, 22 embryos were selected for isolation of RNA, *n* = 6 for days 10, 11, and 12, and *n* = 4 for day 13 (for details, see Table 1). A fraction (200 to 850 μl) of the lysate obtained after homogenization with a buffer containing a nonionic detergent was stored for analysis of the protein content. Total RNA was extracted from the remaining lysate. The concentration and quality of the RNA were assessed with a NanoDrop One (ThermoFischer Scientific) spectrophotometer and an Agilent RNA 6000 Nano assay on an Agilent 2100 Bioanalyzer (Agilent Technologies). The Agilent 2100 Bioanalyzer RNA integrity number (RIN) ranged from 9.1 to 10 (mean = 9.7).

**TABLE 1:** Recovered embryos and their size measured in utero via ultrasound (US) and *ex utero* via ruler

Day p.o.	Mare ID	No. of embryos	Embryo ID	Diameter (mm) embryo 1		Diameter (mm) embryo 2	
				US	Ruler	US	Ruler
10	10	2	E19, E20	nd	3	nd	4
10	11	1	E5	nd	3–4		
10	17	1	E15	4.8	5		
10	18	1	E1	nd	3		
10	23	1	E10	nd	4		
11	11	1	E11	8	10		
11	12	1	E2	nd	2–3		
11	13	2	E6, E7	6.7 × 6.8	7	6.1	7
11	17	1	E21	7.6	9		
11	23	1	E16	5.7	8		
12	16	1	E22	7.3 × 6.8	7		
12	17	2	E17, E18	9.7 × 9.3	10	9.6 × 9.8	11
12	20	1	E3	nd	4		
12	20	1	E8	9.2 × 8.8	9		
12	23	1	E12	10.6 × 9.6	nd		
13	17	2	E13, E14	nd	nd	nd	nd
13	21	1	E4	9.7 × 8.5	11		
13	23	1	E9	nd	8		

Abbreviations: p.o., post ovulation; nd, not determined.

#### 2.2.2 RNA‐Sequencing

RNA‐Seq library preparation was performed starting from 200 ng total RNA using the SEQuoia Complete Stranded RNA Library Prep Kit (Bio‐Rad Laboratories, Inc.), which permits the capture of long as well as short RNAs in a single library. Two pools of 11 samples were prepared, and sequencing was performed on one SP flow cell on an Illumina NovaSeq 6000 instrument (Functional Genomic Center Zurich). Paired‐end sequencing was performed with 92 bp for read one (cDNA insert) and 8 bp for read 2 (UMI sequence for removal of PCR duplicates) and revealed between 24 and 42 million reads per library.

#### 2.2.3 RNA‐seq data analysis

A locally installed version of Galaxy^31^ was used to analyze the resulting sequence reads (FastQ files). Sequencing reads were processed using Cutadapt (Galaxy version 1.16.8) with the parameters—u 1 (trim first base at 5′)−a A{10} (trim any poly[A] track and following bases in the read),−m 15 (removes reads shorter than 15 bases), and a quality cutoff of 28. Trimmed reads were mapped to the current horse genome reference assembly (EquCab3.0) with HISAT2 (Galaxy version 2.1.0 + galaxy4). NuDUP mark/remove PCR duplicates based on molecular tags (Galaxy version 2.3.3) was used to remove PCR duplicates from the BAM files before counting reads mapped to annotated features of the equine genome with the tool feature Counts (Galaxy version 1.6.4 + galaxy1). The latest NCBI GFF3 genome annotation file was used. The obtained read count table was filtered based on counts per million (cpm) cut‐ off 1.15 in at least four samples to remove reads with negligible read counts. A separate counting was performed for reads mapping to microRNAs (miRNAs) with MiRDeep2 Quantifier (Galaxy version 2.0.0) based on equine, bovine, and human miRNA sequences of miRBase (version 22.1). MicroRNAs that showed at least 10 counts in at least 4 out of 5 samples for days 10, 11, and 12 and in at least 3 out of 4 samples for day 13 were used for further differential expression analysis. Further analysis was performed in R with the BioConductor package TCC^32^ to identify differentially expressed genes (DEGs) and miRNAs. In TCC, parameter norm. method = “tmm” was used for normalization and test.method = “edger” for differential gene expression analysis (multiclass design). Genes with a false discovery rate (FDR) <1% were defined as DE. RNA‐seq data have been deposited at NCBI's Sequence Read Archive (SRA) under the BioProject accession ID PRJNA841863 (https://www.ncbi.nlm.nih.gov/sra/PRJNA841863).

### 2.3 Proteome analysis

#### 2.3.1 Sample preparation and processing

Samples were concentrated using 10 K Amicon centrifugal filter devices (Merck). To remove substances interfering with digestion and mass spectrometry, electrophoresis was performed until the samples entered the gel (NuPAGE™ 4%–12%, Thermo Fisher Scientific). Protein containing bands were excised and subjected to in gel digestion. For this, proteins were reduced with 45 mM dithioerythritol in 50 mM NH_4_HCO_3_ for 30 min at 55°C. Subsequently, cysteine side chains were alkylated with 100 mM iodoacetamide in 50 mM NH_4_HCO_3_ in the dark (2 × 15 min). After washing the gel slices with 50 mM NH_4_HCO_3_, an in‐gel digestion with 70 ng LysC (Wako Chemicals) was performed for 4 h at 37°C followed by an overnight digestion with 70 ng trypsin (sequencing grade modified trypsin, Promega). Supernatants were recovered and dried in vacuo (Vacuum concentrator, Bachofer). LC–MS/MS analysis was performed using an Ultimate 3000 nano‐LC system (Thermo Fisher Scientific) coupled to a QExactive HF‐X mass spectrometer (Thermo Fisher Scientific). Peptides were dissolved in 0.1% formic acid and injected to a trap column (Acclaim® PepMap 100, 100 μm × 2 cm, nanoViper C18, 5 μm, 100 Å, Thermo Fisher Scientific). Separation was performed at a flow rate of 250 nl/min using an EasySpray column (PepMap RSLC C18, 75 μm × 50 cm, 2 μm, 100 Å, Thermo Fisher Scientific). As solvent A, 0.1% formic acid was used. The chromatography method consisted of two consecutive gradients from 3% to 25% solvent B (0.1% formic acid in acetonitrile) in 30 min and from 25% to 40% B in 5 min. MS and MS/MS acquisition was performed in cycles of one full scan at 60 k resolution and a maximum of 12 data‐dependent 15 k resolution MS/MS scans. Thermo RAW files were analyzed using MaxQuant (v.1.6.1.0)^33^ with the equine subset of the NCBI RefSeq protein database.

#### 2.3.2 Mass‐Spectrometry data analysis

For protein identification, a FDR < 0.01 at the peptide and protein level was applied. The list of identified and quantified proteins was filtered based on the number of samples per group where the respective protein could be quantified. All proteins which were quantified in at least 3 out of 4 (P10, P11, P12, P13) samples in at least one of the four experimental groups passed the filter. The filtered protein list was used for statistical analysis with BioConductor R package samr (v. 3.0) and proteins with a *q* < 0.05 were considered as differentially abundant proteins (DAP). The mass spectrometry proteomics data have been deposited to the ProteomeXchange Consortium (http://proteomecentral.proteomexchange.org) via the PRIDE partner repository^34^ with the dataset identifier PXD034350.

### 2.4 Data Mining

K‐means clustering was performed to identify clusters of DEGs with similar expression profiles across days after ovulation, while self‐organizing tree algorithm (SOTA) analysis was used to cluster DAP with similar expression profiles (Multiple Experiment Viewer, MeV v.4.8.1, https://sourceforge.net/projects/mev‐tm4/).^35^ Functional annotation analysis was performed using Metascape (www.metascape.org).^36^ miRNA target analysis was performed with MIENTURNET webtool (database miRTarBase) (http://userver.bio.uniroma1.it/apps/mienturnet/)^37^ and with DIANA tools MirPath v.3 (https://dianalab.e‐ce.uth.gr/html/mirpathv3/index.php?r=mirpath).^38^ To identify enriched functional terms for target genes of miRNAs identified in embryos and endometrium, DAVID functional annotation clustering was used (https://david.ncifcrf.gov/).^39^ DAVID functional annotation chart and PANTHER classification system (http://www.pantherdb.org)^40^ was used for protein functional analysis.

## 3 RESULTS

### 3.1 Results of the animal trial and embryo collection

In general, the size (diameter) of the recovered embryos increased with pregnancy day. The highest variation of the size was observed on day 11 post‐ovulation (Table 1). The sizes ranged from 3 to 5 mm on day 10, 2.5–10 mm on day 11, 4–11 mm on day 12, and 8–11 mm on day 13 of pregnancy (see Table 1). For day 13 post‐ovulation, embryos larger than 11 mm were also obtained (data not shown) but not used in this study. For each day of pregnancy, there was a twin pregnancy (recovery of two embryos). In total, 22 embryos were used in this study (day 10 *n* = 6, day 11 *n* = 6, day 12 *n* = 6, day 13 *n* = 4).

### 3.2 The transcriptome profile changes according to the size of the embryo

#### 3.2.1 Identification of dynamic RNA profiles across days 10–13 post‐ovulation

**FIGURE 1:**
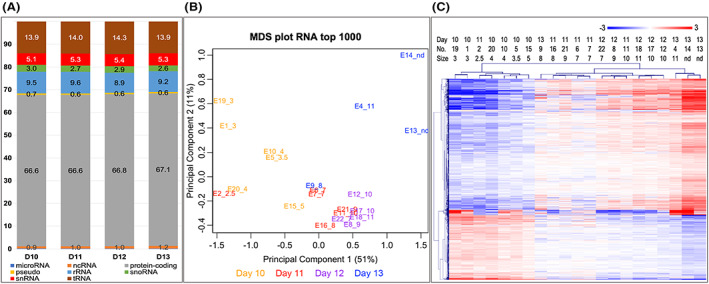
RNA‐seq analysis of embryos across pregnancy days. (A) Distribution (%) of read counts assigned to various classes of RNAs for the days (D) after ovulation. (B) Principal component analysis (EdgeR multi‐dimensional scaling plot, https://bioconductor.org/packages/release/bioc/html/edgeR.html)^102^ of the top 1000 genes with largest variation across the data set. A gradual clustering from left to right in the first dimension depending on the embryo size ranging from 2.5 to 11 mm was found. Sample ID: embryo number and size in mm. (C) Results of unsupervised hierarchical clustering (HCL) created with Pearson correlation coefficient by MeV software (Multiple Experiment Viewer, MeV v.4.8.1, https://sourceforge.net/projects/mev‐tm4/).^35^ HCL of the 1311 DEGs (FDR <1%) confirmed a clustering of the samples according to embryo size. Rows indicate DEG, while columns represent individual embryo samples collected on days 10–13 after ovulation. Mean‐centered expression values (log2 counts per million of a sample—mean of log2 counts per million of all samples) for all embryo samples are shown. Color scale is from −3 (blue, lower than mean) to 3 (red, higher than mean).

A total of 14,140 expressed genes were identified across all samples (Table S1). Among these genes were 12,171 protein‐coding (mRNAs), 273 genes annotated as pseudogenes, 12 ribosomal RNA genes (rRNAs), 461 transfer RNA genes (tRNAs), 259 small‐nucleolar RNA genes (snoRNAs), 82 small nuclear RNA genes (snRNAs), 796 other non‐coding RNA genes (ncRNAs), and 79 miRNA genes. The reproducibility of the library protocol was confirmed by the analysis of the read count distribution to various classes of RNAs. The comparison of the 22 samples showed very low variation in the percentages of different RNA types between the days of pregnancy (Figure 1A) and across individual samples (Figure S1). Moreover, it showed a successful reduction of rRNA fragments (Figure 1A, Figure S1). The highest number of read counts (67%) were assigned to protein‐coding genes.

Principal component analysis (PCA) showed that samples were grouped according to embryo size (principal component 1) (Figure 1B). Besides, similar gene expression for embryos collected on days 11 and 12 was found, except for a very small day 11 embryo and the 4 mm day 12 embryo (the latter excluded from data analysis at this step). Moreover, samples from days 10 and 13 were separated in principal component 2 (Figure 1B). Only one small (8 mm) day 13 and a 5 mm day 10 embryo grouped closer to the day 11 and day 12 embryos with a similar size.

**FIGURE 2:**
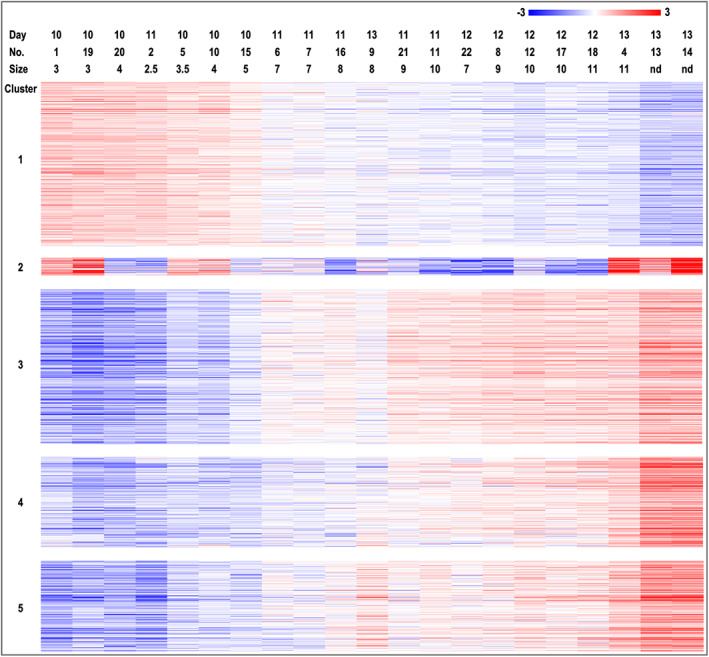
Clustering of DEG with similar expression profiles across pregnancy days and embryo size. K‐means clustering (Multiple Experiment Viewer, MeV v.4.8.1, https://sourceforge.net/projects/mev‐tm4/)^35^ resulted in five clusters with similar expression profiles. Mean‐centered expression values (log2 counts per million of a sample—mean of log2 counts per million of all samples) are shown. Color scale is from −3 (blue, lower than mean) to 3 (red, higher than mean).

Statistical analysis of all identified RNAs across days revealed a total of 1311 DEGs (FDR <1%; Table S2). A hierarchical cluster analysis (HCL) of the DEGs showed clustering of samples according to the conceptus size (Figure 1C). Interestingly, HCL grouped samples into two main clusters: diameter ≤5 mm (small, S embryos) and ≥7 mm (large, L embryos). Additionally, the 1311 DEGs were analyzed for their gene expression profiles across samples using k‐means clustering. This analysis showed that DEGs were grouped into 5 different clusters with 418, 45, 389, 229, and 230 genes in clusters 1, 2, 3, 4, and 5, respectively (Figure 2, Table S1). Clusters 1 and 3–5 showed expression patterns depending on the size of the embryos, while cluster 2 showed a kind of biphasic pattern. Cluster 1 genes showed decreased expression with increasing conceptus size, and clusters 3–5 increasing expression with size. Cluster 3 expression increased already from 7 mm, cluster 5 from 8 mm, and cluster 4 from 9 to 10 mm.

#### 3.2.2 Functional annotation of clusters of DEGs with similar expression profiles

**FIGURE 3:**
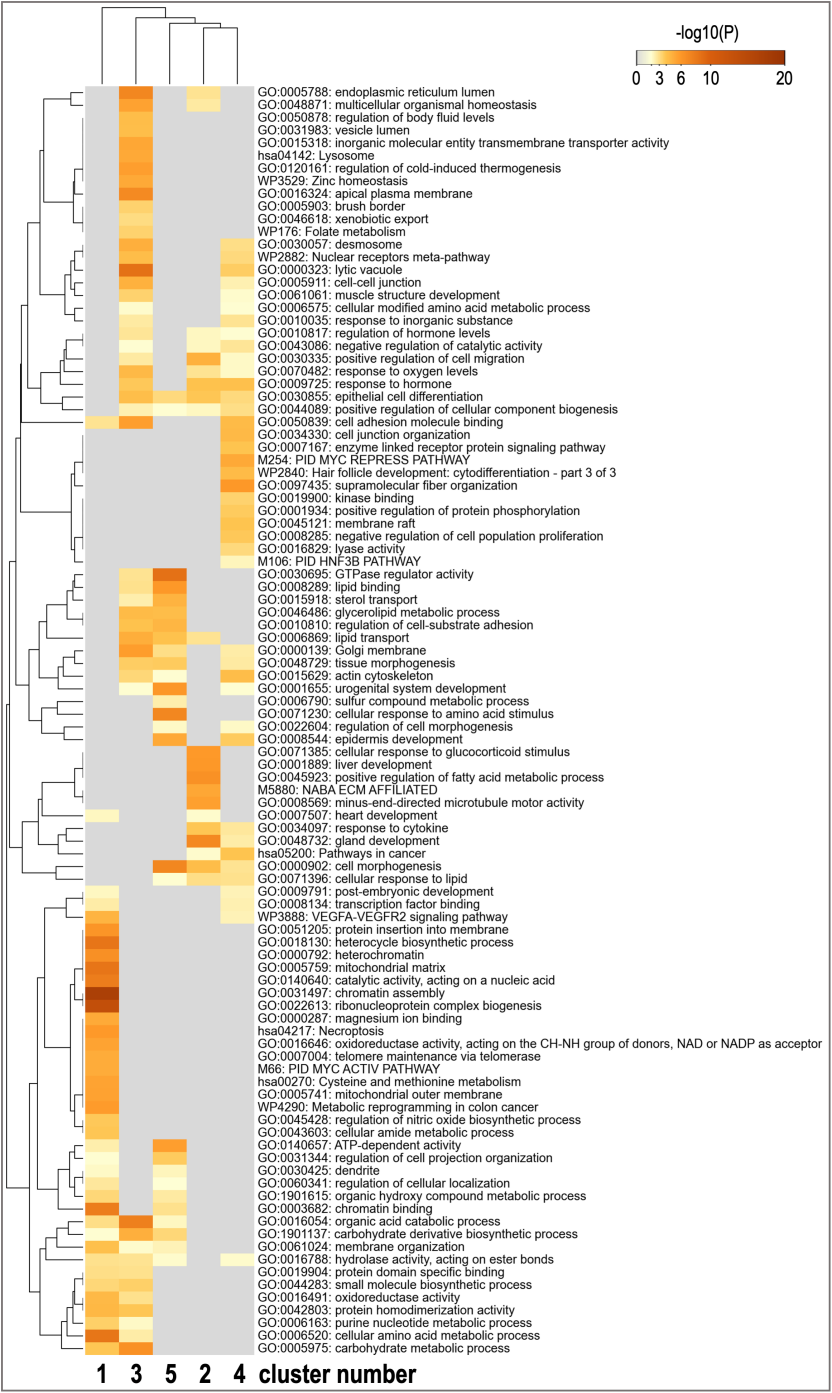
Heatmap of overrepresented GO categories and molecular pathways of the 5 gene expression clusters. The heatmap cells are colored by their *p*‐values from gray (not significant, lack of enrichment) to brown (highly significant). Image created with Metascape webtool (https://metascape.org)^36^ and modified with Adobe Photoshop v.22.4.3.

Figure 3 shows the top 100 enriched functional terms obtained for the 5 clusters by Metascape analysis (https://metascape.org).^36^ The enrichment analysis showed a specific set of biological functions and pathways for each cluster and also some overlap among clusters. Particularly, a higher number of terms were uniquely overrepresented for cluster 1, with terms associated to e.g.: ‘chromatin assembly’ and ‘ribonucleoprotein complex biogenesis’, ‘cellular amino acid metabolic process’. Clusters 2, 3, and 4 also showed uniquely overrepresented terms. Table 2 shows the top 5 overrepresented functional terms of each cluster. Cluster 1 shared enriched functional terms mainly with clusters 3 and 5, but mostly with clearly different significance levels for the enrichment. Cluster 2 shared overrepresented terms with clusters 3–5 with similar significance levels for ‘cellular response to lipid’, ‘response to hormone’, ‘lipid transport’, and ‘epithelial cell differentiation’. Clusters 3 and 4 shared functional terms with similar significance, such as ‘cell adhesion molecule binding’, ‘nuclear receptors Meta‐pathway’, and ‘actin cytoskeleton’. Similar enrichment was found between clusters 3 and 5 for the terms ‘glycerolipid metabolic process’ and ‘regulation of cell‐substrate adhesion’. In addition, clusters 4 and 5 shared ‘epidermis development’ with similar overrepresentation. Details of the enrichment results can be found in Tables S3–S7.

**TABLE 2:** Top five overrepresented functional terms for the five clusters of differentially expressed genes

Term	Cluster 1	Cluster 2	Cluster 3	Cluster 4	Cluster 5
1	Chromatin assembly	Gland development	Lytic vacuole	Supramolecular fiber organization	GTPase regulator activity
2	Ribonucleoprotein complex biogenesis	Positive regulation of fatty acid metabolic process	Organic acid catabolic process	PID MYC repress pathway	Cell morphogenesis
3	Cellular amino acid metabolic process	Liver development	Endoplasmic reticulum lumen	Response to hormone	Cellular response to amino acid stimulus
4	Chromatin binding	Cellular response to glucocorticoid	Apical plasma membrane	Cell junction organization	Urogenital system development
5	Heterocycle biosynthetic process	Minus‐end‐directed microtubule motor activity stimulus	Cell adhesion molecule binding	Actin cytoskeleton	Lipid binding

#### 3.2.3 Identification of dynamic miRNA profiles across days 10 to 13 post‐ovulation

**FIGURE 4:**
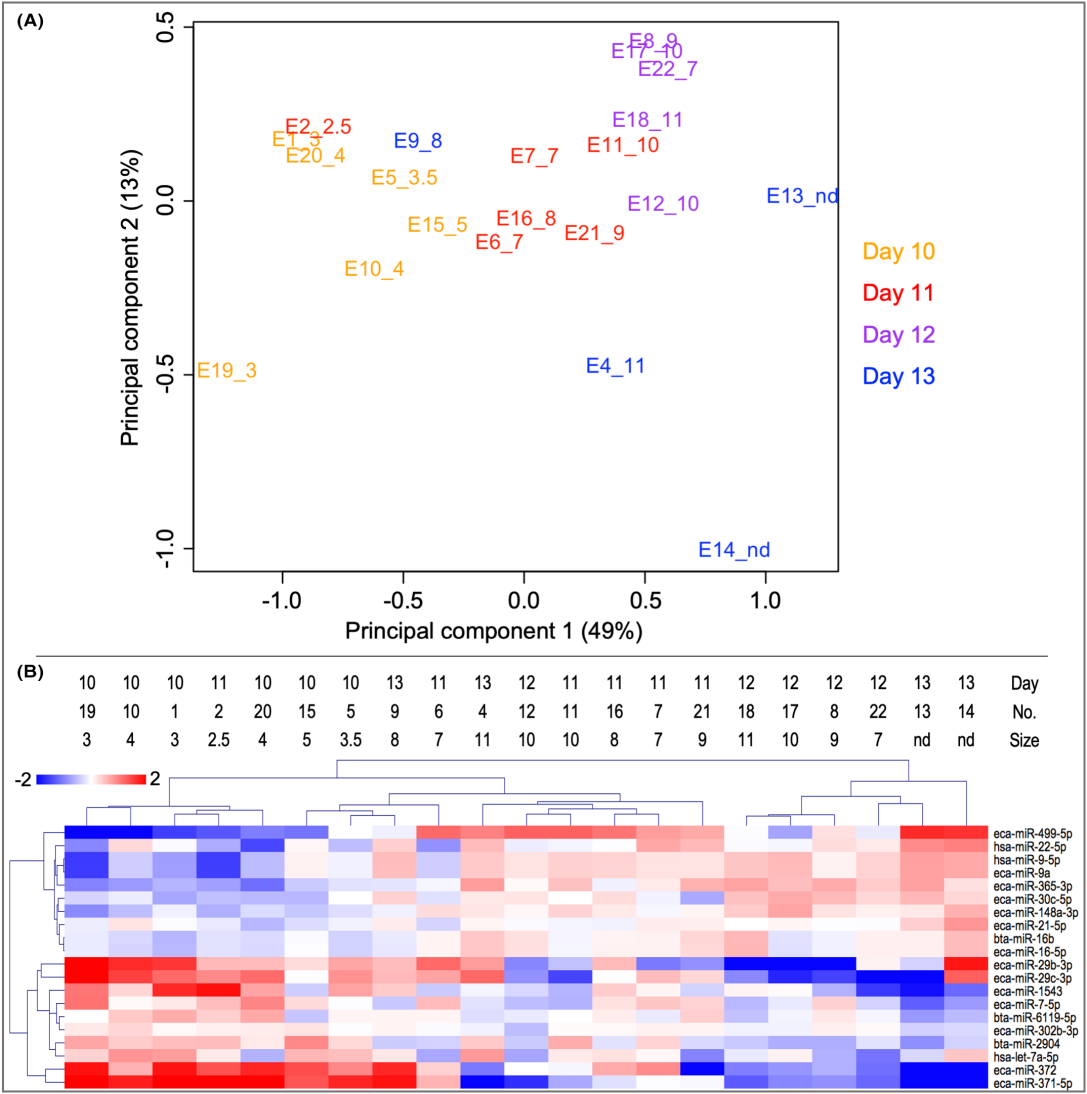
Analysis of microRNA expression across pregnancy days. (A) Principal component analysis (EdgeR multi‐dimensional scaling plot) of the top 20 miRNAs with the highest variation across the data set. Sample ID: embryo number and size in mm. (B) Hierarchical cluster analysis (HCL) of the DE miRNAs (Multiple Experiment Viewer, MeV v.4.8.1, https://sourceforge.net/projects/mev‐tm4/).^35^ Mean‐centered expression values (log2 counts per million of a sample—mean of log2 counts per million of all samples) are shown.

A total of 79 miRNAs were identified across samples (Table S1). A PCA, based on the top 20 miRNAs with the greatest changes across the data set, showed that samples were clearly distributed according to the size of the conceptus (Figure 4A). The conceptus collected on day 13 with a size of 8 mm grouped close to embryos with 3.5–5 mm in size. A similar result was obtained by HCL of the differentially expressed (DE) miRNAs (Figure 4B), which revealed 8 miRNAs as upregulated in conceptuses ≥7 mm (L) and 10 as upregulated in conceptuses ≤5 mm (S) (Figure 4B). Since miRNA mapping and annotation analysis identified miR‐9a and miR‐16b with different names in miRBase for the horse and human or cattle (eca‐miR‐9a and hsa‐miR‐9‐5p; eca‐miR‐16‐5p and bta‐miR‐16b), these miRNAs appeared double reducing the number of DE miRNAs to 18 (Table S2).

#### 3.2.4 Functional annotation of DE miRNAs

Human orthologs of the DE miRNAs were used for further functional annotation analysis with DIANA mirPath v.3 webtool (15 were found out of 18 DE miRNAs) (Table S2). DIANA mirPath v.3 webtool provided overrepresented KEGG pathways for the 7 miRNAs higher in small (Figure S2) and the 8 miRNAs higher in large embryos (Figure S3) (Tables S3 and S4). The most significantly enriched pathways for small embryos were ‘ECM‐receptor interaction’, ‘protein digestion and absorption’, ‘focal adhesion’, ‘lysine degradation’, ‘estrogen signaling pathway’, and ‘TGF‐beta signaling pathway’. For large embryos, the most significantly enriched pathways were ‘fatty acid metabolism’, ‘fatty acid biosynthesis’, ‘biotin metabolism’, ‘ECM‐receptor interaction’, ‘prolactin signaling pathway’, ‘signaling pathways regulating pluripotency of stem cells’, and ‘mucin‐type O‐glycan biosynthesis’.

Further analysis with DIANA mirPath and based on GO biological functions showed that 4 of the miRNAs upregulated in S were associated with GO terms related to embryonic development; ‘post‐embryonic development’ (miR‐372‐3p, miR‐371a‐5p, miR‐302b‐3p), ‘embryonic placenta development’ (miR‐7‐5p), ‘in utero embryonic development’ (miR‐372‐3p, miR‐302b‐3p), ‘embryonic organ morphogenesis’, and ‘embryonic process involved in female pregnancy’ (miR‐371a‐5p). Besides, let‐7a‐5p was linked to ‘response to mechanical stimulus’ and ‘response to prostaglandin F’, miR‐371a‐5p to ‘negative regulation of prostaglandin secretion’, and miR‐29b‐3p/miR‐29c‐3p to ‘negative regulation of prostaglandin biosynthetic process’. In addition, miR‐29b‐3p/miR‐29c‐3p were associated with ‘steroid receptor RNA activator RNA binding’, miR‐371a‐5p with ‘cellular response to steroid hormone stimulus’ and let‐7a‐5p to ‘steroid biosynthetic process’. Furthermore, GO terms related to lipids were obtained, e.g., ‘cellular lipid metabolic process’ (miR‐371a‐5p, miR‐7‐5p, miR‐302b‐3p) and ‘negative regulation of lipid binding’ (miR‐371a‐5p).

Of the miRNAs upregulated in large embryos, 5 were associated with embryonic development or hormone‐related GO terms; ‘post‐embryonic development’ (miR‐30c‐5p), ‘in utero embryonic development’ (miR‐365a‐3p, miR‐30c‐5p, miR‐16‐5p, miR‐9‐5p), ‘embryonic morphogenesis’ (miR‐499a‐5p), ‘prostaglandin J receptor activity’ (miR‐499a‐5p), and ‘cellular response to prostaglandin D stimulus’ (miR‐499a‐5p). Details of GO functions associated with miRNAs for small and large embryos are shown in Tables S5–S8.

#### 3.2.5 Identification of predicted target genes for the DE miRNAs and their potential links with changes in endometrium and embryo

Target gene analysis for the 15 DE miRNAs across samples resulted in 2051 predicted target genes for 7 miRNAs with higher expression in small embryos and 2849 predicted target genes for 8 miRNAs with higher expression in large embryos (source miRTarBase, FDR = 0.3). Considering that the embryo miRNAs could target genes in the embryo but also in the endometrium, the predicted target genes identified for miRNAs upregulated in small (2051) and large embryos (2849) were compared to gene expression changes in the embryos (present study) and the endometrium (DEGs in the LE in comparison of samples derived from pregnant mares and corresponding cyclic nonpregnant controls^23^). Table 3 shows the overlap between the target genes of the DE miRNA for small and large embryos and the DEGs of the endometrial LE and the conceptus. Details of the specific target genes in LE and conceptus can be found in Table S9.

**TABLE 3:** Overlap between the target genes of differentially expressed miRNA in day 10–13 embryos and the DEGs of the endometrial luminal epithelium (LE) (A) and embryos (B, C)

A		DEGs downregulated in LE	DEGs upregulated in LE	Ratio
	Total *n*°	437	690	1.58
		Overlap with miRNA target genes		
miRNA upregulated in >5 mm embryos (2849 target genes)	Total *n*°	103	147	1.43
	hsa‐miR‐148a‐3p	7	22	3.14
	hsa‐miR‐16‐5p	38	69	1.82
	hsa‐miR‐21‐5p	29	38	1.31
	hsa‐miR‐22‐5p	4	7	1.75
	hsa‐miR‐30c‐5p	19	21	1.11
	hsa‐miR‐365a‐3p	2	8	4.00
	hsa‐miR‐499a‐5p	4	2	0.50
	hsa‐miR‐9‐5p	19	23	1.21
miRNA upregulated in <5 mm embryos (2051 target genes)	Total *n*°	53	79	1.49
	hsa‐let‐7a‐5p	21	41	1.95
	hsa‐miR‐29b‐3p	6	13	2.17
	hsa‐miR‐29c‐3p	8	13	1.63
	hsa‐miR‐302b‐3p	10	12	1.20
	hsa‐miR‐371a‐5p	19	19	1.00
	hsa‐miR‐372‐3p	10	11	1.10
	hsa‐miR‐7‐5p	12	14	1.17

B		Downregulated in ≥ 7 mm embryos	Upregulated in ≥ 7 mm embryos	Ratio
	Total *n*°	392	787	2.01
		Overlap with miRNA target genes		
miRNA upregulated in >5 mm embryos (2849 target genes)	Total n°	93	147	1.58
	hsa‐miR‐148a‐3p	11	17	1.55
	hsa‐miR‐16‐5p	53	75	1.42
	hsa‐miR‐21‐5p	14	39	2.79
	hsa‐miR‐22‐5p	3	4	1.33
	hsa‐miR‐30c‐5p	15	13	0.87
	hsa‐miR‐365a‐3p	1	6	6.00
	hsa‐miR‐499a‐5p	7	2	0.29
	hsa‐miR‐9‐5p	11	21	1.91

C		Downregulated in ≤5 mm embryos	Upregulated in ≤5 mm embryos	Ratio
	Total n°	787	392	0.50
Overlap with miRNA target genes				
miRNA upregulated in <5 mm embryos (2051 target genes)	Total *n*°	109	66	0.61
	hsa‐let‐7a‐5p	26	16	0.62
	hsa‐miR‐29b‐3p	28	9	0.32
	hsa‐miR‐29c‐3p	26	13	0.50
	hsa‐miR‐302b‐3p	23	6	0.26
	hsa‐miR‐371a‐5p	16	13	0.81
	hsa‐miR‐372‐3p	27	6	0.22
	hsa‐miR‐7‐5p	30	23	0.77

Further functional annotation enrichment analysis using DAVID functional annotation clustering was performed for these predicted target genes downregulated in LE and conceptus. For potential target genes of miRNAs higher in small embryos, enriched functional terms for LE were related to the regulation of fatty acid metabolism, cell morphology, extracellular exosome, embryo morphology and development, and response to estradiol (Table S10). For potential target genes in the conceptus, enriched functional terms were associated with response to amino acids, focal adhesion, ECM‐receptor interaction, response to nutrients level, starvation, lipids, estradiol, and growth factors, embryo development, gastrulation, and regulation of actin cytoskeleton (Table S11).

For potential target genes of miRNAs higher in large embryos, enriched functional terms for LE were related to regulation of cell communication, response to transforming growth factor‐beta stimulus, stem cell proliferation, regulation of BMP signaling pathway, lipid metabolism, cytoskeleton organization, extracellular exosome, embryonic morphogenesis, embryo development, morphogenesis of embryonic epithelium, immune system development, and steroid metabolic process (Table S12). For potential target genes in the conceptus, enriched functional terms were associated with RNA binding and processing, ncRNA metabolic process, ribosome biogenesis, snoRNA binding, regulation of RNA stability, methylation, DNA packing, histone binding, DNA replication, mitochondrial transport, intracellular protein transport, and regulation of translation (Table S13).

### 3.3 Proteomic profile changes according to embryo size

**FIGURE 5:**
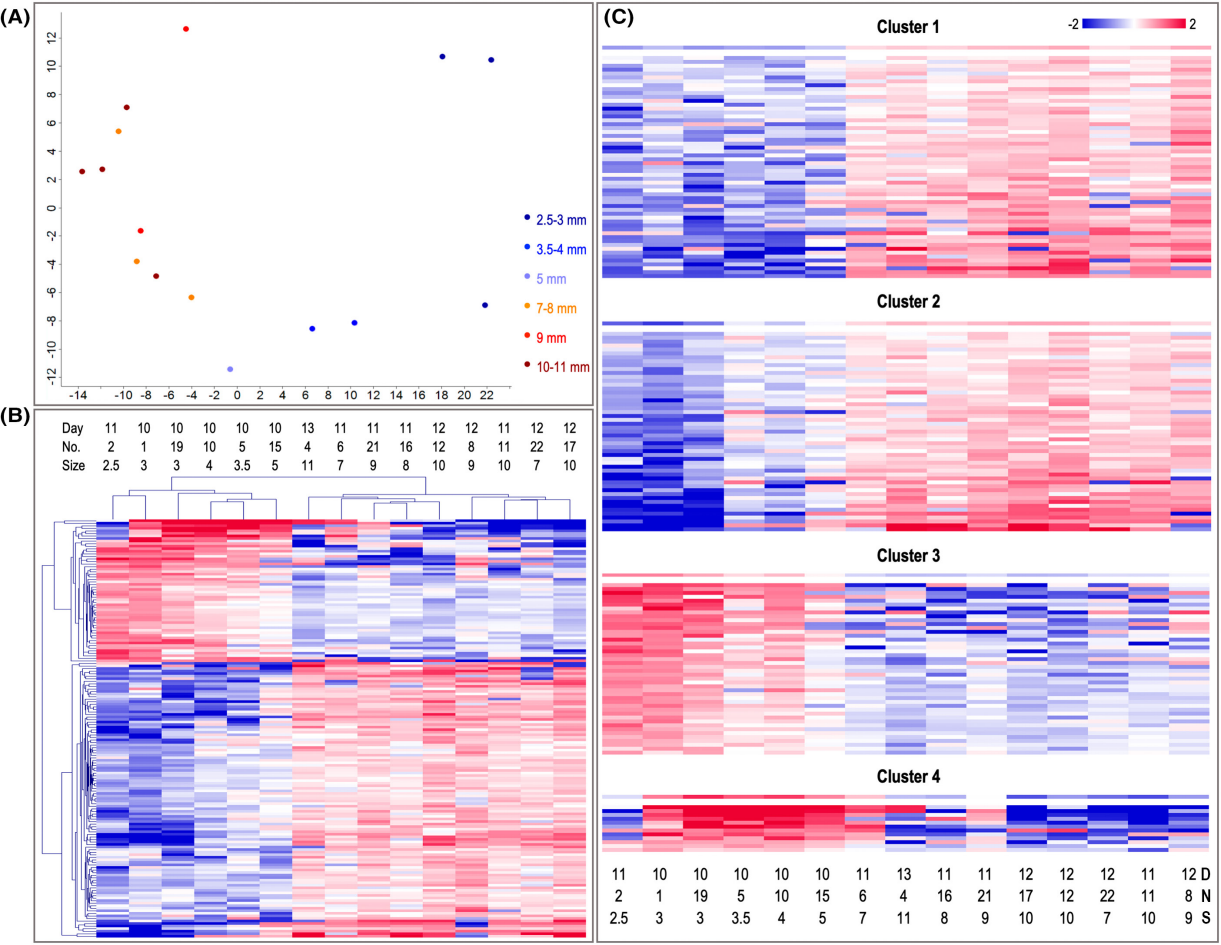
Proteomic analysis of embryos across pregnancy days. (A) Principal component analysis of the data set across embryos collected on days 10, 11, 12, and 13 after ovulation. A gradual clustering from right to left in the first dimension depending on the size ranging from 2.5 to 11 mm was observed. (B) Unsupervised hierarchical clustering (HCL) analysis created with Pearson correlation coefficient by MeV using 164 differentially abundant proteins (DAP) having a fold change (FC) ≤ − 1.5 or ≥1.5 between small (≤5 mm, S) and large (≥7 mm, L) embryos. Rows indicate DAP, while columns represent individual embryo samples. Mean‐centered expression values (log2 counts per million of a sample—mean of log2 counts per million of all samples) are shown. Color scale is from −2 (blue, lower than mean) to 2 (red, higher than mean). (C) Clusters of DAP with similar profiles across pregnancy days and embryo size. SOTA clustering analysis (Multiple Experiment Viewer, MeV v.4.8.1, https://sourceforge.net/projects/mev‐tm4/)^35^ was performed and resulted in 4 clusters with similar expression profiles. Mean‐centered expression values (log2 counts per million of a sample—mean of log2 counts per million of all samples) are shown. Color scale is from −2 (blue, lower than mean) to 2 (red, higher than mean). (D) day; N: embryo number; S: size/size in mm.

A total of 2200 proteins were identified across samples (Table S1). PCA showed a clear distribution mainly according to the embryo size (Figure 5A). Conceptuses collected on day 10 and with sizes between 2.5 to 5 (small embryos) separated mainly in principal component 1. Embryos collected on days 11–13 and with sizes between 7 and 11 (large embryos) did not show such a clear separation and separated more in principal component 2. After filtering and statistical analysis, 259 proteins were found as differentially abundant (DA) between large and small embryos, 107 proteins downregulated and 152 proteins upregulated in large versus small embryos (Table S2). DA proteins (DAPs) displaying a fold change (FC) ≤ −1.5 or ≥1.5 (164 proteins) were used for further HCL clustering analysis (Table S3). HCL grouped samples into two main clusters, DAPs increased in small or large embryos (Figure 5B). Additionally, the 164 DAPs were analyzed for their expression profiles across samples using SOTA clustering analysis. This analysis provided four different clusters with similar protein expression depending on the size of the embryo, with 57, 51, 44, and 12 proteins in clusters 1, 2, 3, and 4, respectively (Table S4). Cluster 1–2 proteins showed increased expression with increasing conceptus size, while clusters 3–4 represented decreasing expression with increased size. Despite the high similarity of clusters 1 and 2, the expression of proteins in cluster 2 already seemed to increase in conceptuses of ≥3.5 mm slightly. Cluster 4 contained proteins whose expression levels appeared to be more dependent on the day post‐ovulation than the embryo size, with the highest expression on day 10 and lowest on day 12.

#### 3.3.1 Functional annotation of DAPs clusters with similar expression profiles

**FIGURE 6:**
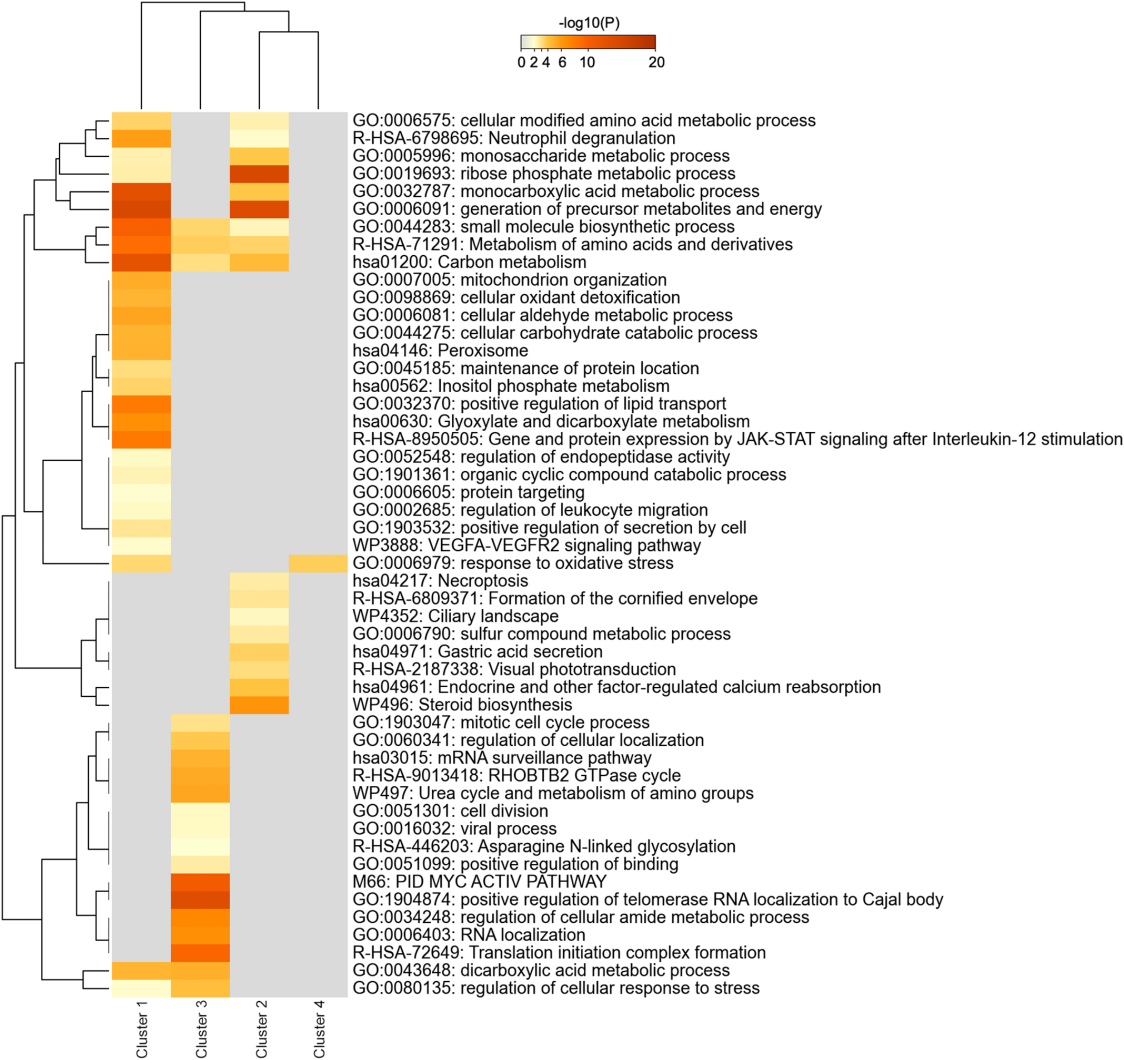
GO categories and molecular pathways of the 4 protein expression clusters. The heatmap cells are colored by their *p*‐values from gray (not significant, lack of enrichment) to brown (highly significant). Image created with Metascape webtool (https://metascape.org)^36^ and modified with Adobe Photoshop v.22.4.3.

Metascape analysis provided functional terms and pathways associated with the proteins in each SOTA cluster (Figure 6) (Table S5). Figure 6 shows that each cluster resulted in a specific set of enriched functional terms with a small overlap among clusters. Cluster 1 proteins were related to ‘monocarboxylic acid metabolic process’, ‘positive regulation of lipid transport’, and ‘gene and protein expression by JAK‐STAT signaling after interleukin‐12 stimulation’. Cluster 2 proteins were related to ‘steroid biosynthesis’ and ‘ribose phosphate metabolic process’. Cluster 3 proteins were explicitly enriched for ‘translation initiation complex formation’, ‘PID MYC activ pathway’, and ‘positive regulation of telomerase RNA localization to the Cajal body’. Cluster 4 proteins were only enriched for ‘response to oxidative stress’, which was shared with proteins of cluster 1. An overlap of enriched functional terms between clusters 1, 2, and 3 was observed for ‘small molecules biosynthetic process’, ‘metabolism of amino acid and derivates’, and ‘carbon metabolism’.

The analysis of all identified proteins using the PANTHER classification system and DAVID analysis (Gene Ontology main category cellular component) revealed that 44% and 24% of the proteins, respectively, belonged to the extracellular region (potentially secreted). Furthermore, all proteins and DAP were compared to proteins identified in equine uterine extracellular vesicles (uEVs) found in a parallel study,^41^ revealing an overlap of 9% of proteins between embryo and uEVs (Table S6).

### 3.4 Integration of embryo, uEVs, and endometrial datasets

The same embryo samples used for proteomic analysis (15 embryos) were also used in the transcriptomic analysis. Integration of the proteomic and transcriptomic datasets (2200 proteins and 14,061 RNAs identified) showed that 95% of the proteins were also identified at the mRNA level. The remaining 101 proteins which did not overlap with the mRNAs were compared to the uEVs protein cargo dataset,^41^ and about 68% of these proteins were found in uEVs. Further comparison between all proteins identified in the embryo and proteins identified in corresponding uEVs showed that almost half of the embryo proteins were also found in uEVs. Examples of these proteins which could be secreted into the uterine fluid by the embryo in part via EVs or be derived from the endometrium in part via endometrial EVs were: molecules involved in prostaglandin synthesis and signaling (AKR1A1: aldo‐keto reductase family 1 member A1, AKR1B1: aldo‐keto reductase family 1 member B, PLA2G2A: phospholipase A2 group IIA, PTGES3: prostaglandin E synthase 3, PTGFRN: prostaglandin F2 receptor inhibitor), in the transport of nutrients (APOA1: apolipoprotein A1, APOE: apolipoprotein E, LCN2: lipocalin 2, P19: P19 lipocalin or uterocalin), 10 Solute Carrier (SLC) family A members, and cell adhesion (FGA: fibrinogen alpha chain, FN1: fibronectin 1, ITGB1: integrin subunit beta 1, MUC4: mucin 4, cell surface associated, SPP1: secreted phosphoprotein 1, also known as osteopontin). Moreover, other growth factor‐related proteins (CCN2: cellular communication network factor 2, IGFBP2: insulin like growth factor binding protein 2; IGFBP7: insulin like growth factor binding protein 7, INHBA: inhibin subunit beta A, INHBB: inhibin subunit beta B), galectin 1 (LGALS1, high mRNA expression in embryo, protein expression increased in embryo with growth and detected in uEVs), and the chemokine CCL28.

Furthermore, the comparison of the DEGs (1311) and DAPs (259) identified across embryo samples resulted in 42 genes that were DE at both protein and mRNA levels. Of the DAPs with FC cut‐off = 1.5 (164 proteins), 35 proteins were overlapping (Table S7). Further comparisons were performed between the 5 clusters of DEGs with similar expression profiles and the 4 clusters of DAPs, i.e., for clusters with similar up‐ or downregulation in small or large embryos, respectively (Table S8). For higher expression in small embryos, DEG cluster 1 was compared to DAP clusters 3 and 4. This resulted in an overlap of 12 genes. For higher expression in large embryos, DEG clusters 3–5 were compared to DAP clusters 1–2. This resulted in an overlap of 19 genes (Table S8).

Additionally, potential upstream regulators (EGF: epidermal growth factor, IGF1: insulin like growth factor 1, TGFB1: transforming growth factor beta 1, TNF: tumor necrosis factor, VEGF: vascular endothelial growth factor, IFNG: interferon gamma, IL1B: interleukin 1 beta, and IL6: interleukin 6)^23^ identified in a recent study in the equine endometrium during days 10–13 of pregnancy were looked up in the lists of mRNAs and proteins detected in the embryo. In the embryo, expression of EGF receptor mRNA was found but not for *EGF*. The mRNA of IGF1 was detected in the embryo but also in the endometrium. Expression of *TGFB1* and *TGFB2*: transforming growth factor beta 2 as well as their receptors, was detected in endometrial LE and GE and in the embryo. The uEVs also contained TGFB2 protein. Expression of *TNF* was not detectable, but considerable amounts of TNF superfamily member 10 (*TNFSF10*) mRNA, also known as *TRAIL*, were found in the endometrium but not in the embryo. TNFSF10 protein was identified in uEVs. Vascular endothelial growth factor A (*VEGFA*) and vascular endothelial growth factor B *(VEGFB)* mRNA were detected in the embryo. The concentration of *VEGFA* increased with embryo growth. In the endometrium, VEGFA, VEGFB, and VEGFC (vascular endothelial growth factor C) (not in GE) expression were identified.

The expression of *IFNG* and *IL6* was not detectable in the embryo, endometrium, or in uEVs. Expression of IL1B was also not detected in the embryo and only at low levels in endometrial LE and ST. The only interleukin with a considerable expression level in the embryo was interleukin 17F (*IL17F*). The *IL17F* mRNA expression increased with embryo growth, with the highest levels on day 12 after ovulation (12‐fold higher compared to day 10). The protein could also be detected in the embryo. The expression of *IL17F* was not detectable in the endometrium.^23^ Interleukin 17 receptor A (*IL17RA*), interleukin 17 receptor D (*IL17RD)*, and interleukin 17 receptor E (*IL17RE*) were also expressed in the embryo. The interleukin 17F (IL17F) receptor mRNAs *IL17RA*, *IL17RC* (*i*nterleukin 17 receptor C), *IL17RD*, *IL17RE* were detected in the endometrium.^23^

## 4 DISCUSSION

The findings of the present study revealed an exciting and dynamic transcriptomic and proteomic profile of the horse embryo from 10 to 13 days of gestation, i.e., a time coinciding with MRP. Interestingly, the size of the embryo rather than the gestational age was the primary determining factor of these numerous specific temporal changes. During days 10–13 of pregnancy, the equine embryo is characterized by rapid expansion,^42^ which was reflected in our study by an increasing size from 3 mm on day 10 to more than 11 mm on day 13 of gestation. This daily growth of about 3 mm/day is consistent with reported values in ultrasonography studies.^4^

Interestingly, two distinct expression profiles were found, one with increased expression in small embryos (2.5–5 mm) and the second with increased expression in large embryos (7–11 mm). Some sub‐profiles could be identified, which mainly differed in the embryo size when the expression started to increase. These RNAs or proteins with increased expression in large embryos (particularly>10 mm) might play an essential role in MRP signaling. The overrepresentation of functions related to processes in the nucleus, e.g., ‘DNA packaging’, ‘gene silencing’, and ‘cell cycle’ for mRNAs and proteins increased in small embryos, and mRNAs and proteins increased in large embryos related to ‘cellular response to extracellular stimulus’, ‘cell–cell communication’ or ‘metabolism of lipids’ was in agreement with observations reported by a previous study analyzing day 8 vs. day 10–14 equine embryos, but without considering embryo size.^26^

Equine blastocyst size varies mainly with age of the embryo 8–9 days post‐ovulation.^43^ In our study, the sex of the embryo identified based on genes linked to X and Y chromosomes did not seem to affect the size (data not shown), which is supported by a previous study.^43^ However, this warrants further investigations involving a large number of female and male embryos where a large number of embryos of the same size had to be collected. Overall, to the best of our knowledge, there are no studies showing that slight differences in embryo size affect the transcriptome and proteome of the equine embryo during the period of investigation herein.

### 4.1 Genes and proteins with a potential key role during MRP

#### 4.1.1 Synthesis and metabolism of steroid hormones

As the equine embryo is capable of steroidogenesis from around day 6,^12^ various DEGs identified in this study in equine embryos were associated with responding to steroid hormones and steroid and sterol metabolic processes. Also, a great variety of DAPs and DEGs was related to steroid and sterol metabolic processes and regulation of hormonal levels (Reactome pathway Metabolism of steroids; https://reactome.org/PathwayBrowser/#/R‐HSA‐8957322&PATH=R‐HSA‐1430728, R‐HSA‐556833). At the mRNA level, *HSD3B2* (hydroxy‐delta‐5‐steroid dehydrogenase, 3 beta‐ and steroid delta‐isomerase 2; involved in P4 synthesis), *CYP11A1* (cytochrome P450 family 11 subfamily A member 1), and *STAR* (steroidogenic acute regulatory protein) were found highly expressed in large embryos, particularly day 13 embryos. Similarly, a study reported a higher expression of *STAR* and *CYP11A1* in day 12 and 14 embryos when compared to day 8.^26^ The concentration of the enzymes cytochrome P450 family 17 subfamily A member 1 (CYP17A1; P4 to 17alpha‐OH‐progesterone), CYP19A1 (aromatase, cytochrome P450 family 19 subfamily A member 1), and HSD17B1 (estrogen activation, hydroxysteroid 17‐beta dehydrogenase 1) was increased in large embryos. These results are consistent with previous studies showing an increase in steroid synthesis, especially of E2 with developmental stage.^44^ By contrast, *HSD17B14* (hydroxysteroid 17‐beta dehydrogenase 14), involved in the metabolism of steroids and converting E2 into estrone (E1),^45^ was found as downregulated both at mRNA and protein levels with increase in embryo size. In this line, it has been reported that interconversion of E2 and E1 in the equine trophoblast varied with developmental stage, favoring E2 in older embryos.^46^

#### 4.1.2 Prostaglandin synthesis, metabolism, and signaling

The production of PGE2 (prostaglandin E2) and PGF2α (prostaglandin F2α) by the equine embryo has been associated with embryo mobility, and likewise with maintenance of an appropriate uterine PGE2:PGF2α ratio responsible for prevention of luteolysis. Different genes and proteins belonging to the phospholipase A2 (PLA2) group of enzymes were found in the embryo; worth noting PLA2 plays a role in providing arachidonic acid as a precursor of prostaglandin synthesis, by mediating its release from cell membrane phospholipids. Particularly, *PLA2G7* (phospholipase A2 group VII) and *PLA2G12B* (phospholipase A2 group XIIB) mRNA expression was higher in large embryos compared to small embryos (mainly on day 13). By contrast, *PLA2G4A* (phospholipase A2 group IVA) expression was similar across days 10–13, in agreement with a previous study.^47^ At the protein level, PLA2G2A and PLA2G7 were detected with high intensity values for PLA2G2A which was not detectable at mRNA level. Since it has been shown that distinct PLA2 enzymes regulate PGE2 (PLA2G4C: phospholipase A2 group IVC) and PGF2α (PLA2G6: phospholipase A2 group VI) production in bovine endometrial epithelial cells,^48^ it is likely that distinct PLA2 enzymes could likewise modulate the prostaglandin (PG) production in the equine embryo.

Expression of *PTGS1* (prostaglandin‐endoperoxide synthase 1, also known as COX‐1) and *PTGS2* (prostaglandin‐endoperoxide synthase 2, also known as COX‐2) was identified at the mRNA levels in all embryos on day 10–13 and for *PTGS2* also at the protein level. Both genes encode central enzymes of PG synthesis, converting arachidonic acid into prostaglandin H2 (PGH2), which is further metabolized to PGE2 and PGF2α. However, no differences were found between small and large embryos. Based on the read count data, expression of *PTGS2* was higher compared to *PTGS1* in the embryo regardless of the size which is consistent with a previous study.^47^ Additionally, all three PGE2 synthases, *PTGES* (membrane mPGEs‐1), *PTGES2*, and *PTGES3* (both cytosolic PGEs) were similarly expressed at the mRNA level and were proposed as the main players in the two independent PGE2 production systems.^47^ Only at the protein level, the membrane‐located PTGES showed increased expression in large embryos, suggesting that in large embryos the glutathione‐dependent PGE2 production is more important. PTGES has been found as colocalized and functionally coupled with PTGS2 and PTGS1 in the perinuclear envelope and was markedly induced by proinflammatory stimuli.^49^

The synthesis of PGF2α in the embryo could be from PGE2 as previously suggested via carbonyl reductase [NADPH] 1 (CBR1).^47^ The *CBR1* mRNA was detected at low expression levels and decreasing with embryo growth. Besides, our results identified relatively high expression of mRNA and protein of *AKR1A1* (aldo‐keto reductase family 1 member A1) and *AKR1B1* (aldo‐keto reductase family 1 member B1), which have been associated with significant prostaglandin F synthase activity,^50^ proposing that these genes are responsible for PGF2α synthesis in the equine conceptus. Furthermore, in human and porcine endometrium, AKR1B1 has been shown to be the primary PGF2α synthase.^51^, ^52^ Whereas *AKR1B1* mRNA and protein were slightly decreased in day 13 embryos, *PTGFR* (prostaglandin F receptor, encoding for the receptor of PGF2α) mRNA expression was increased with embryonic growth. In addition, *PTGFRN* (prostaglandin F2 receptor inhibitor) mRNA was highly expressed on all days studied and found at protein level, confirming the recent identification of PTGFRN protein in the blastocoel fluid of day 10 equine embryos.^30^ Since none of the PGE2 receptors was detectable as expressed in the equine embryo, the results obtained suggest that PGE2 produced by the embryo is mainly acting on the endometrium while PGF2α affects both the embryo and endometrium.

Several candidates for transporters involved in PG export were also expressed in the embryonic samples. Of these, *ABCC2* (ATP binding cassette subfamily C member 2) showed increased mRNA expression with increased embryo size and has been described to export prostaglandins.^53^ A related transporter, ABCC6 (ATP binding cassette subfamily C member 6), showed a very similar expression pattern, but the identity of the in vivo substrates of this transporter remains elusive.^54^ Since PGs derived from the endometrium are also important for embryonic growth and development,^55^ the increased expression in large embryos of *SLCO2A1* (solute carrier organic anion transporter family member 2A1), which is mediating the cellular import of PGs and thromboxanes,^56^ suggests that this gene as involved in this process.

**FIGURE 7:**
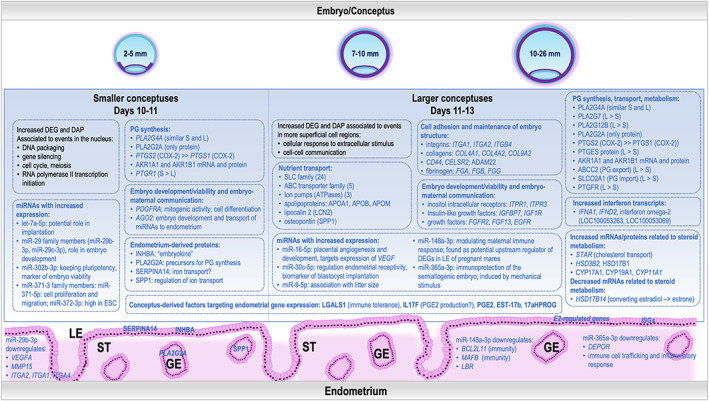
Schematic illustration of the main transcriptomic and proteomic findings in equine embryos collected on days 10, 11, 12, and 13 after ovulation and selected interactions with the maternal endometrium. LE: luminal epithelium; GE: glandular epithelium; ST: stroma.

Furthermore, the expression of *PTGR1* (prostaglandin reductase 1) mRNA encoding an enzyme involved in PG degradation^57^ was found as decreasing with embryonic growth. In contrast, PTGR1 protein has been detected in higher amounts in the uterine fluid of pregnant mares when compared to cyclic mares on day 13 of gestation,^27^ and higher mRNA expression was found in endometrial LE of pregnant mares on (days 10–13) compared to cyclic non‐bred control mares.^23^ These findings suggest that PG degradation is primarily located in the endometrium and uterine fluid but not in the embryo. The complex expression of changes related to PGs are summarized in Figure 7.

#### 4.1.3 Interferon‐related genes

In contrast to ruminants, where interferon tau represents the main signal for MRP,^58^ the role of interferons in the horse is still ambiguous, being only a few of them detected as expressed in equine embryo around the time of MRP.^26^ One study reported that neither alpha or omega interferons were detected in the horse embryo,^59^ while a second study reported the absence of interferon gamma expression in horse trophoblasts.^60^ Conversely, another study reported very low expression levels of interferons α1, δ, and ω2, between days 10–14 and the expression substantially increased on day 16 of pregnancy, after the onset of MRP.^61^ Our study confirmed the latter findings, and also detected the low expression of two interferon omega‐2 genes (*LOC100053263*, *LOC100053069*), two interferon‐delta genes (*IFND1*, *IFND2*), and 5 interferon alpha genes (*IFNA1*, *IFN‐ALPHA2*, *LOC100053110*: interferon alpha‐4, *LOC100053210*: interferon alpha‐2‐like, *LOC100052921*: interferon‐alpha‐4), and mainly increasing with embryonic growth. The finding that several typical interferon‐stimulated genes (ISGs), e.g., *MX1*: MX dynamin like GTPase 1, *MX2*: *MX dynamin like GTPase 2*, *OAS1*: 2′‐5′‐oligoadenylate synthetase 1, *OAS2*: 2′‐5′‐oligoadenylate synthetase 2, *OAS3*: 2′‐5′‐oligoadenylate synthetase 3, *IFIT1*: interferon induced protein with tetratricopeptide repeats 1, *IFI6*: interferon alpha inducible protein 6, and *IFI27*: interferon alpha inducible protein 27 were found as expressed in LE on days 10–13 post‐ovulation and some of them with higher expression in pregnant compared to cyclic non‐bred mares^23^ suggests that some of the interferons detected in the equine embryo have an effect on the endometrium.

#### 4.1.4 Nutrient transport

As the nutrition of the preimplantation equine embryo relies solely on the histotroph, the embryo transfers the nutrients from the uterine environment through different transporters to support its needs. Various transporters have been identified in the equine embryo^26^, ^62^, ^63^ and uterine secretome.^30^, ^64^ In agreement, our study identified 24 transporters of the SLC family, 5 members of the ATP‐binding cassette (ABC), 3 ion pumps (ATPases), 3 apolipoproteins (*APOA1*: apolipoprotein A1, *APOB*: apolipoprotein B, *APOM*: apolipoprotein M) and P19 lipocalin. The expression profiles of some transporters were exceptionally dynamic, while others remained steady, reflecting the needs of the embryo as it develops. In the case of P19, which provides the equine embryo with lipids,^64^ a dynamic profile at mRNA level was found, with highest expression at day 10, followed by a marked decrease on days 11 and 12 and slightly increasing again on day 13 of gestation, in line with a previous study.^26^ In contrast, at protein level, a more constant profile was found without differences regardless embryo size or pregnancy day. This is in line with our results derived from uEVs,^41^ where no differences for P19 at mRNA and protein levels were found across pregnancy days. Furthermore, this is also in line with our concurrent study of the endometrium^23^; where no differences across pregnancy days were observed in the GE, where *P19* was highly expressed. Overall, the low expression of P19 in the equine embryo when compared to uEVs^41^ and endometrium,^23^ further supports the maternal origin of P19 protein and mRNA, and suggests the transport of P19 to the embryo via EVs.

#### 4.1.5 Embryonic development and viability

Intracellular receptors 1 and 3 for inositol 1,4,5‐trisphosphate (*ITPR1* and *ITPR3*) were identified in higher concentrations in large compared to small embryos. A recent study showed that *ITPR1* and *ITPR3* are essential for fetal‐maternal signaling and embryo viability.^65^ Genes associated to the insulin like growth factor system (*IGFBP7*, *IGF1R*: insulin like growth factor 1 receptor) and fibroblast and epidermal growth factor family (*FGFR2*: fibroblast growth factor receptor 2, *FGF13*: fibroblast growth factor 13, and *EGFR*: epidermal growth factor receptor) also showed a higher gene expression in large embryos. The increased expression of fibroblast growth factor (FGF) signaling members may influence the equine embryo itself, by promoting endoderm formation.^66^ A study in human trophoblast showed that the low‐affinity IGF‐binding protein IGFBP7 may play a key role in regulation of E2‐induced trophoblast proliferation and invasion.^67^

In contrast, platelet derived growth factor receptor alpha mRNA (*PDGFRA*), which has a high mitogenic activity and is involved in the control of cell differentiation, was more abundantly expressed in small embryos. Murine studies showed that timing and expression level of Pdgfra are crucial for embryo development and affects multiple endoderm and mesoderm derived structures.^68^

### 4.2 MicroRNAs and their potential roles in embryo development and MRP

Despite the increasing evidence of the role of miRNAs as key regulators of gene expression during pregnancy and particularly in embryo development,^69^, ^70^, ^71^, ^72^ little is known about miRNA profiles in the equine embryo. Only one study has shown the miRNA profile of day 13 equine embryos.^19^ The present study provided the first miRNA signature of the equine embryo from day 10 to day 13, showing a very fine‐tuned regulation according to its size. Moreover, our results proposed specific miRNAs and their different target genes in the embryo and endometrium, as potential regulators of MRP signaling events.

#### 4.2.1 MicroRNAs increased in small embryos

In mice, suppression of let‐7a expression in the embryo increased the number of implantation sites which probably was mediated by upregulation of Igf1, Itgb3 (integrin subunit beta 3), and Tgfb1.^73^ Furthermore, mucin 1, which inhibits attachment of the embryo to the endometrium, has been shown to be a direct target of let‐7a indicating a positive effect of let‐7a on implantation in the endometrium. In this sense, our results in the equine embryo (lower let‐7a levels in large embryos) suggest downregulation of let‐7a as potentially involved in delayed implantation in the horse. The higher concentration of miR‐29b‐3p in small embryos agrees with findings in murine embryos where miR‐29b is important for preimplantation embryo development by controlling a network of genes which includes reprogramming factors and molecular regulators of post‐transcriptional modification processes.^74^ Likewise, miR‐302‐3p could be important in early embryo development according to its role related to pluripotency.^75^ Moreover, miR‐372 has been identified as highly expressed in human embryonic stem cells and decrease of expression with embryonic development soon after cell differentiation,^76^ which is in line with our results. For miR‐371‐5p, promotion of proliferation, migration, and invasion of choriocarcinoma cells has been shown, suggesting downregulation of this miRNA in the context of regulation of proliferation, migration, and invasion processes in trophoblast cells.^77^

#### 4.2.2 MicroRNAs increased in large embryos

Genetic studies showed an implication of miR‐499a‐5p in recurrent pregnancy loss in woman.^78^ Furthermore, miR‐16‐5p has been shown to regulate placental angiogenesis and development by targeting expression of VEGF,^79^ suggesting together with its relatively high expression in equine uEVs^41^ a regulatory effect on the endometrium. In cultured bovine embryos, elevated miR‐30c‐5p levels were associated with decreased developmental competence.^80^ Expression in trophectoderm cells of human blastocysts showed positive correlation with embryo implantation.^81^ In this context, our results suggest that miR‐30c‐5p could act upon the endometrium regulating its receptivity for the embryo. Similarly, miR‐9‐5p could be involved in regulation of embryo development and/or endometrial receptivity based on the results of a genetic study in goats showing a strong association with the number of fetuses^82^ and its expression in porcine endometrium during implantation.^83^, ^84^ For miR‐148a‐3p, a regulatory role for immune response in bovine endometrium by reducing inflammatory responses has been shown.^85^ In our previous study,^23^ miR‐148a‐3p was identified among the potential upstream regulators of the DEGs in LE of pregnant mares (conceptus stage corresponding to large embryos in the present study) compared to nonpregnant cyclic mares. Similarly, miR‐365 has been suggested to be involved in regulation of placental development and immunoprotection by targeting the non‐classical HLA class‐Ib molecule HLA‐G (major histocompatibility complex, class I, G).^86^ Moreover, a study in chondrocytes revealed that miR‐365 expression is mechanoresponsive and regulates differentiation of chondrocytes.^87^ These previous findings and the increase of miR‐365‐3p expression with embryonic growth suggests the possibility of induction by the mechanical stimulus of the migration through the uterine lumen which is probably increasing with the embryonic growth.

### 4.3 Integration of embryo, endometrium, and uterine EVs data to unveil key factors during the period of MRP

The data integration suggested that most of the embryonic proteins that were not detected at RNA level were probably taken up from the uterine fluid, given the much higher sensitivity of RNA‐seq. The overall high overlap between proteins identified in the embryo and corresponding proteins found in the uEVs suggested that some of these molecules could be secreted into the uterine fluid by the embryo via EVs while others could be derived from endometrial EVs and transferred to the embryo, given the lack of mRNA expression in the embryo.

In this sense, the protein expression of PLA2G2A in the embryo (not detectable at RNA level) indicated that PLA2G2A was taken up by the conceptus from uEVs probably derived from the endometrial GE (*PLA2G2A* mRNA expression GE > > LE > ST).^23^ The function of PLA2G2A in the embryo could be in lipid metabolism, particularly providing precursors for PG synthesis. Osteopontin (SPP1) showed a similar expression pattern, likewise, suggesting origin from endometrial GE. Originally, SPP1 was mainly thought to be involved in conceptus attachment to the uterine wall and placentation.^88^ The results obtained here suggest a different function in the horse during the investigated time of pregnancy which could be related to the recent finding for SPP1 in the porcine placenta where it binds to integrins and increases ion transport.^89^ Moreover, the two members of the TGF‐beta superfamily INHBA and INHBB seemed to be secreted by endometrial LE and GE and to function as an embryokine as suggested by Kannampuzha‐Francis and others, who found pro‐developmental actions of INHBA in bovine preimplantation embryos.^90^

Several members of the serpin protein family were only detectable in the embryonic samples at the protein level. The uterine SERPINA14 (serine peptidase inhibitor clade A, member 14) has been suggested to have species‐specific functions including modulation of the maternal immune system and in the pig iron transport to the fetus.^91^ The presence of high SERPINA14 concentrations and the strong increase as the embryo growths in the horse herein suggest a similar function as found in pigs, but an effect on the expression of cytokines by the equine embryo could also be possible. The second serpin highly abundant in the equine embryo but decreasing with growth was SERPINB11 (serpin family B member 11). Although, the specific function of this protein is not known, it has been suggested to play an important role in embryonic implantation and decidualization in mice based on its expression pattern in the endometrium and in the trophoblast.^92^

The much stronger expression of *LGALS1* mRNA in the embryo compared to endometrium and high protein expression in embryo and uEVs suggests a regulatory effect of embryo‐derived LGALS1 on the endometrium. Wilsher and others proposed a protective role of LGALS1 expressed in the equine trophoblast from the maternal immune system.^93^ This is also supported by a recent study in cattle where conceptus LGALS1 has been suggested to confer maternal‐conceptus immune tolerance.^94^

The integration of the expression patterns of potential upstream regulators for DEGs identified in the endometrial LE during days 10–13 of pregnancy (EGF, IFNG, IGF1, IL1B, IL6, TGFB, TNF, VEGF)^23^ in embryo, endometrium, and uEVs indicated an effect of endometrial‐derived EGF on the embryo and endometrial LE. The observed expression of *IGF1* did not support its role as important conceptus‐derived factor. The finding that the uEVs contained TGFB2 protein indicates a role in conceptus‐endometrium interactions. As in the pig, TGFB could play a role in embryonic growth and development.^95^ The endometrial TNFSF10 could target the conceptus via uEVs. Based on the proposed functions in other species, this gene could also play a role in establishing an immune privileged environment at the maternal‐embryo interface in the mare.^96^

The only interleukin expressed in the conceptus in considerable amounts was IL17F. The absence of *IL17F* expression in the endometrium but the expression of its high‐affinity receptor IL17RC^97^ in the endometrium (not in the embryo), supports a specific effect of embryo‐derived IL17F on the endometrium. In contrast to IL17A (interleukin 17A), IL17F is also produced by epithelial cells and has been shown to induce different responses in target cells than IL17A.^98^ For example, in chondrocytes, a stimulatory effect of IL17F on *PTGS2* expression and PGE2 production has been shown.^99^ It could be speculated that IL17F has a similar effect on the endometrial epithelium on prostaglandin production involved in MRP which needs to be further investigated.

A good number of the genes identified as DE in the conceptus at protein and mRNA level were associated to steroid synthesis, histotrophic nutrition, endometrial receptivity, and immune response, which could also modulate key events involved in MRP. For example, increased *SCP2* (sterol carrier protein 2) mRNA and protein expression in large embryos might contribute to steroid synthesis by serving as conductor for transporting cholesterol into the outer mitochondrial membrane, after which, cholesterol is imported into mitochondria via the STAR protein (also increased in large embryos) for further steroid synthesis.^100^ SCP2 was previously identified in day 15 pig conceptus.^101^ Other enzymes associated to steroid metabolic processes, CYP17A1, CYP19A1, HSD17B1, and HSD17B4, were found as increased in large embryos.

Overall, the reported transcriptomic and proteomic alterations in the equine embryo, and particularly genes and proteins with increased expression in day 12–13 embryos, confirmed our hypothesis that a combination of signaling molecules or other signals is more likely than a unique signal required for MRP in the horse. Our results provided new insights into the regulation of gene expression in the equine embryo in relationship to endometrial gene expression and uEVs molecular cargo, which represents a strong molecular basis for further studies of potential main factors of MRP in the mare.

## 5 CONCLUSION

This study revealed dynamic transcriptomic and proteomic profiles of the equine embryo during the time of MRP, which are rather depending on the embryo size than day of pregnancy. Overall, genes with increased expression in small embryos were associated to functions in maintenance of cell pluripotency, while genes with increased expression in large embryos were linked to cell‐to‐cell communication, response to extracellular stimulus, and fetal growth. Our results provided new insights to prostaglandin synthesis by the equine embryo and potential miRNAs targeting the embryo itself and the endometrium, which might modulate the maternal immune response, endometrial receptivity, and contribute to the late implantation in the horse. The integration with data derived from uEVs and endometrial LE and GE revealed specific mRNAs, proteins, and miRNAs, particularly those increased in large embryos on days 12–13 of pregnancy, as potential key players in embryonic development, signaling to the endometrium during the MRP, and successful establishment of pregnancy.

## AUTHOR CONTRIBUTIONS

A.R.V., C.A. and S.B.: Conceptualization; A.R.V., C.A., I.C., G.P., H.B.; T.F., and S.B.: Methodology; A.R.V., C.A. and S.B.: formal analysis; A.R.V., C.A. and S.B.: investigation; C.A. and S.B.: resources; A.R.V. and C.A.: writing‐original manuscript; C.A. and S.B.: writing, reviewing and editing; C.A. and S.B.: supervision; C.A. and S.B.: project administration; C.A., H.B. and S.B: funding acquisition. All authors have read and agreed to the published version of the manuscript.

## FUNDING INFORMATION

This research was funded by SNF grant 31003A_173171 and Foundation Pro Pferd (Zurich), Project 2018‐03.

## CONFLICT OF INTEREST

The authors declare no conflict of interest.

## ETHICS STATEMENT

The study was carried out in compliance with the ARRIVE guidelines (https://arriveguidelines.org/) and conform to Directive 2010/63/EU.

## Supporting information


Appendix S1

Figure S1

Figure S2

Figure S3
