# Supplementary figures and images for: Dynamic regulation of the transcriptome and proteome of the equine embryo during maternal recognition of pregnancy
Source: FASEB Bioadv. 2022 Oct 18;4(12):775–97. doi: 10.1096/fba.2022-00063 (PMC9721094; doi:10.1096/fba.2022-00063)

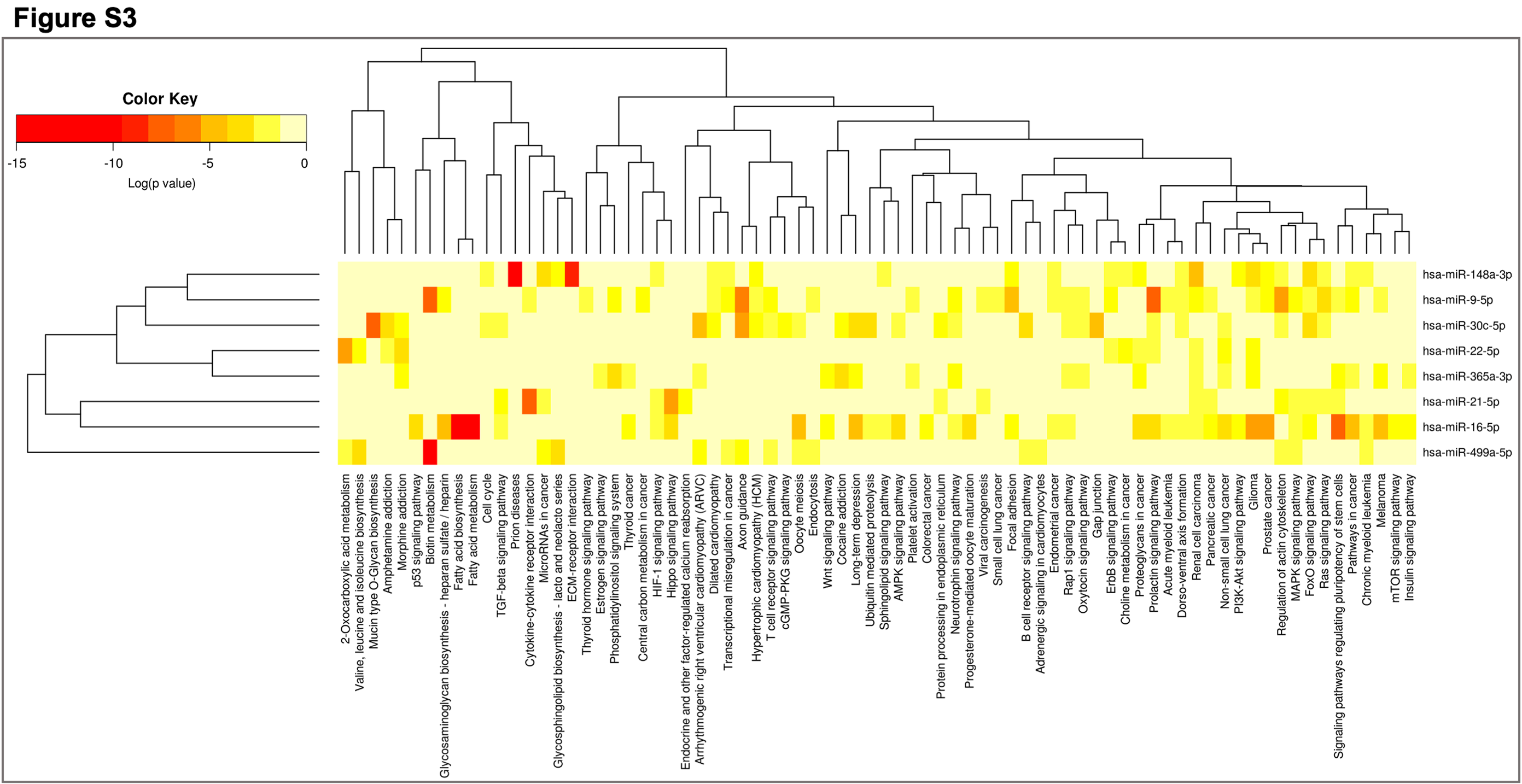

Supplement: Supplementary file 2 — Figure S1 [file FBA2-4-775-s003.tif]

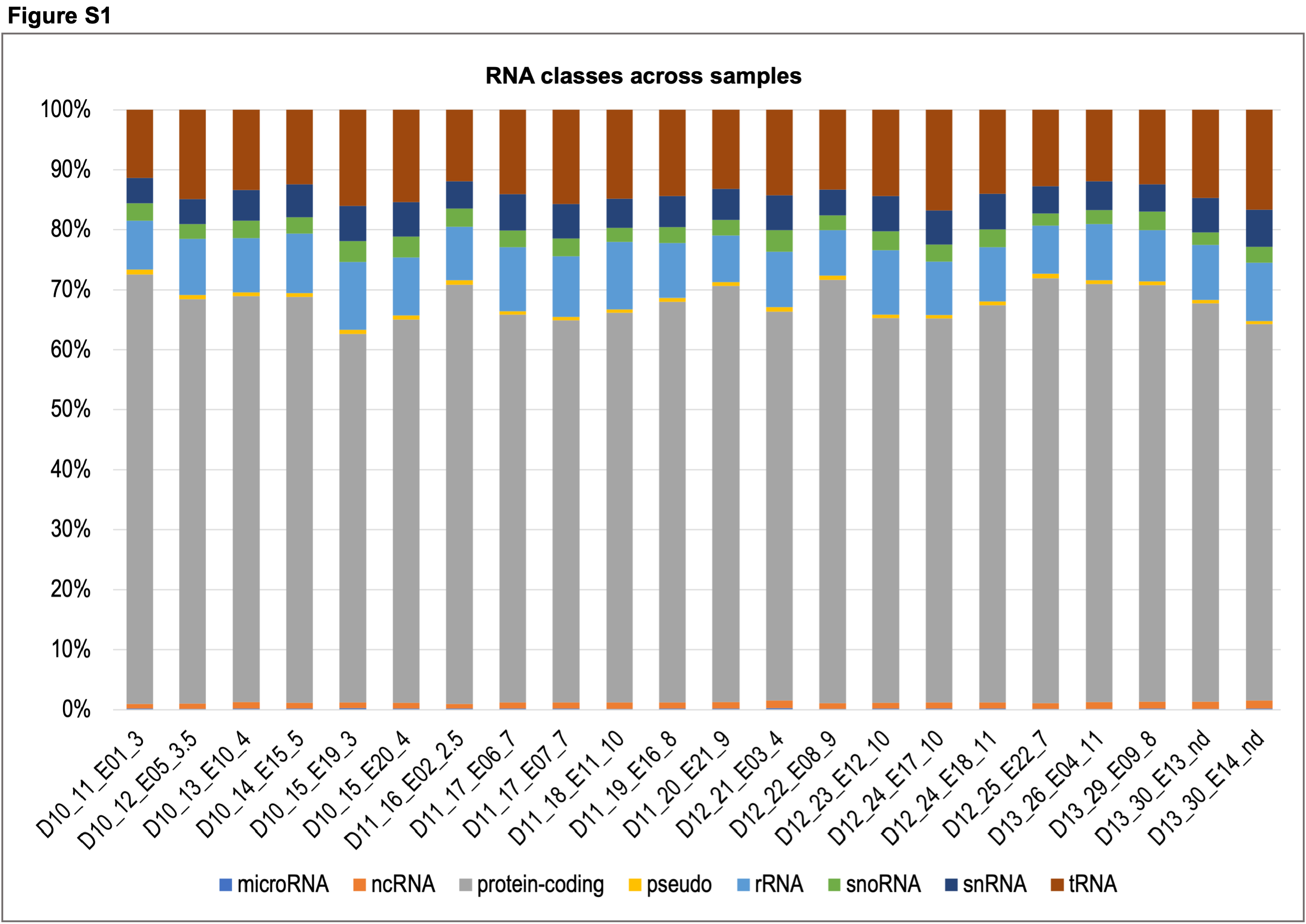

Supplement: Supplementary file 3 — Figure S2 [file FBA2-4-775-s001.tif]

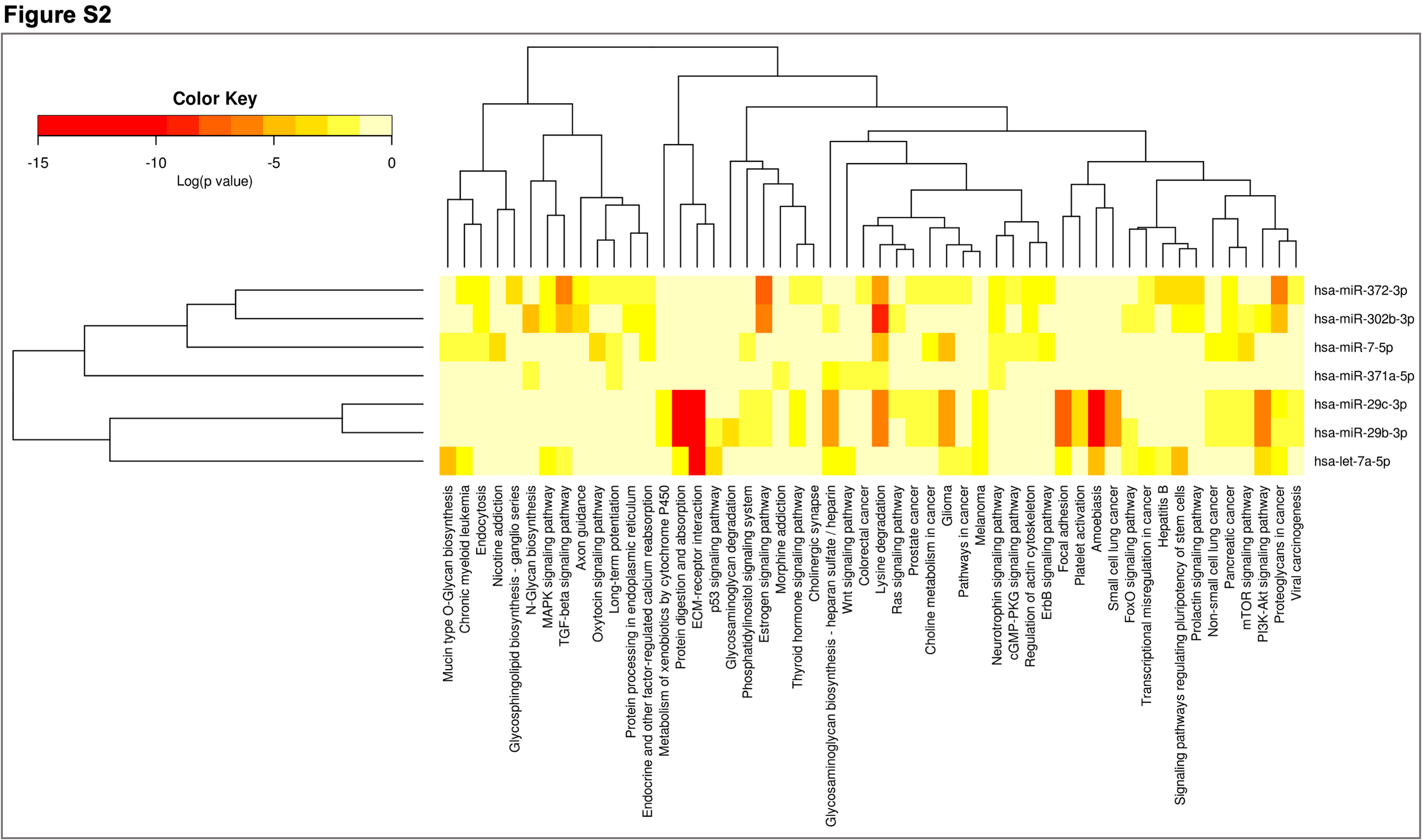

Supplement: Supplementary file 4 — Figure S3 [file FBA2-4-775-s002.tif]
